# Conversation in small groups: Speaking and listening strategies depend on the complexities of the environment and group

**DOI:** 10.3758/s13423-020-01821-9

**Published:** 2020-10-13

**Authors:** Lauren V. Hadley, William M. Whitmer, W. Owen Brimijoin, Graham Naylor

**Affiliations:** Hearing Sciences - Scottish Section, School of Medicine, University of Nottingham, New Lister Building Level 3, Glasgow Royal Infirmary, 10-16 Alexandra Parade, Glasgow, G31 2ER UK

**Keywords:** Conversation, Extra-linguistic behaviour, Speaking and listening, Adverse, Conditions

## Abstract

Many conversations in our day-to-day lives are held in noisy environments – impeding comprehension, and in groups – taxing auditory attention-switching processes. These situations are particularly challenging for older adults in cognitive and sensory decline. In noisy environments, a variety of extra-linguistic strategies are available to speakers and listeners to facilitate communication, but while models of language account for the impact of context on word choice, there has been little consideration of the impact of context on extra-linguistic behaviour. To address this issue, we investigate how the complexity of the acoustic environment and interaction situation impacts extra-linguistic conversation behaviour of older adults during face-to-face conversations. Specifically, we test whether the use of intelligibility-optimising strategies increases with complexity of the background noise (from quiet to loud, and in speech-shaped vs. babble noise), and with complexity of the conversing group (dyad vs. triad). While some communication strategies are enhanced in more complex background noise, with listeners orienting to talkers more optimally and moving closer to their partner in babble than speech-shaped noise, this is not the case with all strategies, as we find greater vocal level increases in the less complex speech-shaped noise condition. Other behaviours are enhanced in the more complex interaction situation, with listeners using more optimal head orientations, and taking longer turns when gaining the floor in triads compared to dyads. This study elucidates how different features of the conversation context impact individuals’ communication strategies, which is necessary to both develop a comprehensive cognitive model of multimodal conversation behaviour, and effectively support individuals that struggle conversing.

## Introduction

In order to understand communication in everyday life, we need to investigate speaking and listening in ecologically valid (Verga & Kotz, 2019), interactive (De Jaegher, Di Paolo, & Gallagher, 2010) situations. Extensive work on the use of language has shown that linguistic behaviour is affected by both the environment (Chambers, Tanenhaus, Eberhard, Filip, & Carlson, 2002) and the interaction situation (Isaacs & Clark, 1987), with theories of language-as-action acknowledging the impact of both physical and social context on speech (Clark, 1996; Irvine et al., 1992). But conversation, the primary site of language use, includes a range of communicative behaviours aside from linguistic content. In this paper, we test whether and how people change their intelligibility-optimising extra-linguistic behaviours according to features of the physical and social context. Specifically, we investigate whether intelligibility-optimising speaking and listening behaviours increase as the acoustic environment and interaction situation becomes more challenging.

Communication involves the complex interplay of perception and production across a range of modalities (Holler & Levinson, 2019). Intelligibility, defined as the extent to which the speaker’s signal is understood by the listener, is dependent on both linguistic and extra-linguistic behaviours (Anderson, Bard, Sotillo, Newlands, & Doherty-Sneddon, 1997; Sumby & Pollack, 1954). The mutuality model of communication (Lindblom, 1990), proposes a complementarity between the speech signal and alternative channels of information (originally termed ‘signal-independent information’). In other words, as intelligibility of speech decreases, alternative factors become more important. While one such factor discussed by Lindblom is the shared knowledge between interlocutors, an alternative way to facilitate intelligibility would be the use of extra-linguistic communicative behaviour.

A range of different extra-linguistic behaviours are engaged during conversation to improve intelligibility. Several such behaviours have been identified through work investigating how people behave in background noise, which reduces intelligibility and increases auditory and cognitive processing difficulty (Heinrich, 2020). From the speaker’s perspective, a broad field of work has shown that as noise increases in level, people speak more loudly (Junqua, Fincke, & Field, 1999), an effect that is amplified in interactive situations (Garnier, Henrich, & Dubois, 2010; Jokinen, Remes, & Alku, 2016), and that provides benefit to listeners (Summers, Pisoni, Bernacki, Pedlow, & Stokes, 1988). Furthermore, increases in noise level have been linked to changes in the duration of utterances, which lengthen in constrained tasks such as path-finding puzzles (Beechey, Buchholz, & Keidser, 2018), but shorten in less constrained tasks such as free conversation (Hadley, Brimijoin, & Whitmer, 2019). Though longer utterances could reflect a floor-holding strategy and facilitate communication by increasing the time available for the listener to decode the message, shorter utterances could reflect talkers choosing to convey less information when sharing becomes challenging. While it may be possible to disentangle these functions by combining utterance duration analyses with word-level analyses (cf., Hansen & Varadarajan, 2009), in this paper we focus on utterance duration alone as an automatically detectable nonverbal behaviour that does not require linguistic analysis. Switching to the listener’s perspective, as noise increases, listeners can increase signal strength both by orienting their ear more optimally (Grange et al., 2018) as well as by moving closer to the talker (Pearsons, Bennett, & Fidell, 1977).

Older adults find everyday conversation to be challenging (Pichora-Fuller, 1997), due to poorer sensory and cognitive processing of linguistic content (Kozou et al., 2005; Pichora-Fuller & Singh, 2006). They report particular difficulty in noisy environments and larger groups (Heinrich et al., 2016; Vas, Akeroyd, & Hall, 2017), leading to the possibility that intelligibility-optimising behaviours in such situations would particularly benefit them. As communication difficulty has been linked to loneliness and negative health outcomes in the ageing population (Palmer, Newsom, & Rook, 2016), understanding the behaviours that older adults use (and do not use) to reduce difficulty in conversation is critical for developing effective support (e.g., through interventions or technology). In this paper we therefore investigate how difficulty communicating due to the complexity of the acoustic context, and the complexity of the interaction context, impact older adults’ use of these extra-linguistic behaviours.

In terms of the complexity of the acoustic context, it is not only the level of the background noise that impacts communication difficulty, but the type of background noise. Most prior work has investigated speaking and listening against a background of steady-state, often speech-spectrum-shaped noise. While this is simple to characterise, it is not necessarily representative of the richness of noise experienced in the real world. Multi-talker babble is a more complex form of background noise with a similar frequency spectrum compared to speech-shaped noise, but that introduces the additional challenge of differentiating linguistic content between target and masker (Freyman, Balakrishnan, & Helfer, 2004). This additional complexity leads to word recognition being poorer in babble than speech-shaped noise (Ezzatian, Li, Pichora-Fuller, & Schneider, 2015), particularly in older adults (Rajan & Cainer, 2008), and also leads to more disrupted neural processing of speech (Kozou et al., 2005). We therefore compare conversation behaviour not only in different levels of noise, but between speech-shaped and babble noise, hypothesising that facilitatory extra-linguistic behaviours will be used to a greater extent in the more complex and challenging conditions, i.e., in louder environments, and in babble rather than speech-shaped noise.

In terms of the complexity of the interaction context, size of conversation group has a substantial impact on communication difficulty. In a conversation, talkers and listeners alternate in quick succession, with only a few hundred milliseconds between utterances (Stivers et al., 2009). This is much less than the time required to plan an utterance (Levinson & Torreira, 2015), indicating that interlocutors predict when their partner(s) will finish. In larger groups individuals must not only predict the current talker, but other listeners as well (who may compete for the next turn), and switching attention between talkers is highly demanding (Lin & Carlile, 2015), particularly for older adults (Getzmann, Hanenberg, Lewald, Falkenstein, & Wascher, 2015). Hence while many studies investigating multi-person interaction have studied dyads, the dyad has been argued to be conceptually unique (Moreland, 2010; though see Williams, 2010) due to the single focus of attention (the partner), the simple alternation of turns (with little negotiation required), and the minimal set of possible roles (i.e., talker vs. addressee). Transitioning to groups such as triads introduces the need to spread attention between interlocutors and negotiate with other waiting listeners for turns, dramatically increasing complexity. We therefore compare conversation behaviour in dyad and triad groups, hypothesising that facilitatory extra-linguistic strategies will be used to a greater extent in the more complex triad than dyad condition.

In sum, we address the understudied effect of context on extra-linguistic behaviour (Patterson, 2013). We specifically test how complexity of the conversation context, and hence difficulty communicating, impacts use of intelligibility-optimising extra-linguistic behaviours. We focus on older adults, who are likely to experience particular difficulty, and manipulate both physical context (noise level and type), and social context (conversation group size). We use the mutuality model of communication to hypothesise that greater difficulty attaining intelligibility will lead to increased reliance on alternative strategies (i.e., more optimal vocal level, utterance duration, head orientation, and distance between interlocutors). Note that as conversational difficulty relates to age, we also present exploratory analyses in which we split relatively younger from relatively older participants, to address whether effects are specific to the older group within our sample.

## Method

### General design

Both the dyad and triad studies reported here involved interlocutors engaging in a series of approximately 10-min-long conversations (trials), while a ring of eight loudspeakers encircling the participants presented background noise. Only one type of background noise was used per conversation, but the levels of that type of noise were varied within each conversation. Prior to the experiment, participants were strangers to each other, and were matched within dyads/triads on better ear average hearing threshold (measured without hearing aids), hearing asymmetry, and age. No participants overlapped between the dyad and triad studies, but participant samples were matched between experiments on better ear average hearing threshold (mean dyads = 22 dB hearing loss (HL), triads = 26 dB HL), hearing asymmetry (mean dyads = 5 dB HL, triads = 4 dB HL) and age (mean dyads = 61 years, triads = 61 years). Any participants with hearing aids were instructed to remove them prior to the experiment in order to avoid hearing-aid signal processing altering the stimulus in uncontrollable ways (Naylor & Johannesson, 2009). All participants were able to understand speech in noise unaided, as verified using a digit-triplet speech-recognition test (Vlaming, MacKinnon, Jansen, & Moore, 2014; dyads M = -18.1 dB signal-to-noise ratio (SNR); triads M = -17.1 dB SNR). Details of the dyad study were previously published in Hadley et al. (2019), but further detail of the triad study, and of differences between the dyad and triad studies, is provided below.

### Triads

The following refers specifically to the triad experiment.

#### Participants

Thirty-three unacquainted native Glaswegian participants were recruited from the participant pool at Hearing Sciences – Scottish Section. This is a pool of predominantly older adults, from which participants with severe hearing or visual difficulties are excluded, as are participants aged over 75 years who have not participated in studies in our lab over the last 2 years. They were divided into 11 mixed-gender triads (age M = 61 years, SD = 11 years, better-ear four-frequency pure-tone average M = 26 dB HL, SD = 10 dB HL). Within triads, participants were matched approximately on age (difference within triad M = 11 years, SD = 10 years), hearing loss (difference within triad M = 17 dB HL, SD = 9 dB HL), and hearing asymmetry (difference within triad M = 6 dB HL, SD = 2 dB HL). Of the triad participants, 14 had normal hearing ( < 25 dB HL), and 19 had mild hearing loss (25–40 dB HL); 14 of the triad participants normally wore hearing aids. Participants were paid £10 for taking part. Ethics approval was obtained from the West of Scotland Research Ethics Committee (09/S0704/12). All experimental conditions were completed in one visit.

#### Materials and task

Triads were seated in an equilateral triangle (1.5 m between each chair) in the centre of a ring (diameter 3.6 m) of eight equidistantly spaced loudspeakers (Tannoy VX-6) situated in a sound-deadened room of 4.3 m × 4.7 m × 2.6 m. Noise was presented continuously (no gaps between levels) at 54, 60, 66, 72 or 78 dB. Noise varied between levels every 20–30 s, and the order of levels was determined using a paired de Bruijn sequence whereby each level was presented once following each other level (individually sequenced for each conversation). To avoid a startle response in participants, smoothing was applied for 10 ms between level changes, and in total, each conversation lasted up to 13 min. Two different noise types were used: speech-shaped noise (eight uncorrelated noise signals) shaped to the long-term average spectrum of Byrne et al. (1994), and eight-talker babble generated by concatenating sentences from Stacey and Summerfield (2007) and presenting four male talkers and four female talkers, all with British accents (one talker per loudspeaker, position randomised in each trial). All triads experienced two conversations in speech-shaped noise, and two conversations in eight-talker babble (order of noise types counterbalanced across trials). Data were pooled across the two conversations in each noise condition.

Head motion was recorded using Vicon Tracker software, sampling at 100 Hz and with spatial resolution of under 0.01° (from eight Vicon Bonita B-10 cameras). Head position was recorded at the centre of the head and in relation to the centre of the room. Eye movement was also recorded using Pupil Labs binocular eye trackers, but technical issues mean that these data will not be reported here. Speech was recorded using a head-worn gooseneck microphone approximately 6 cm from the participant’s mouth. Audio was run at 16 bits and 44.1 kHz sample rate, I/O was handled with a Ferrofish A-16 driven by an RME MadiFace XT on the host computer. MATLAB was used to determine loudspeaker output, record motion capture data, and trigger changes in the presentation level of the background noise.

#### Procedure

Participants were introduced and seated in the lab, before being fitted with motion-tracking crowns, microphones and eye trackers. Hearing aids were not worn during the experiment. Each triad then held four conversations. The conversation topics focused on: film preferences (Rimé, 1982), close-call incidents (Bavelas, Coates, & Johnson, 2000), the resolution of an ethical dilemma (Healey, Purver, King, Ginzburg, & Mills, 2003), and how Glasgow has changed over the years. Order of conversation topics was counterbalanced, and participants were told to try to continue conversing the entire trial regardless of noise level.

### Comparison data for dyads

Details of a prior dyad experiment were reported in Hadley et al. (2019). In the dyad experiment, head movement, eye movement and speech parameters were recorded during conversations in different levels of noise. Similar to the triad experiment, participants were matched according to hearing loss and age, were unfamiliar prior to the study, and received the same instructions regarding conversing in noise levels that varied frequently between 54, 60, 66, 72 and 78 dB. Participant samples were similar across both experiments (i.e., both had the same mean age and similar levels of hearing loss). Of the dyad participants, 19 had normal hearing ( < 25 dB HL), nine had mild hearing loss (25–40 dB HL), and two had moderate hearing loss (41–55 dB HL); eight of the dyad participants normally wore hearing aids. Key differences of the dyad experiment compared to the triad experiment were the use of only one noise type (speech-shaped noise), and the use of only three conversation trials (using the discussion of film, a close-call incident, and an ethical dilemma, but not the discussion of Glasgow).

### Analyses

Data from head movements (in terms of x, y and z coordinates, and yaw, pitch and roll orientations) and data from speech recordings were combined into a single 100-Hz time-aligned array. This array included movement and speech information of all participants referenced to the corresponding background noise level. From these data, participants’ distances from their partners, and orientations toward their partners, were also derived. Note that the triads were positioned in an equilateral triangle, meaning that the angle between a participant looking to the partner to their left and the partner to their right would be 60^o^ (though whether this angle would be positive or negative would depend on their position in the motion-capture space). Thus, for orientation angle relative to the talker, all triad listeners’ head-angle data were transformed so that the talker at any given moment was at 0^o^ and the other listener at approximately 60^o^. Speech level was calculated as the A-weighted root-mean-square amplitude at the centre of the room of each recording in 10-ms segments. The time that each participant was speaking was derived using a simple bespoke algorithm that applied a manually set level threshold on a rolling 100-ms Hanning window of the speech level data. In order not to artificially segment ongoing speech turns, pauses of up to 1.25 s within one person’s speech were allowed due to prior work finding longer gaps to be rare (Campione & Véronis, 2002). In other words, if somebody was silent for less than 1.25 s before continuing to speak, the entire period was recorded as speech. For analysis, speaking was therefore defined as all times when the individual’s microphone signal detected vocal activity, and listening as times when another participant’s microphone signal (but not the individual’s own) detected vocal activity. A speaking turn was defined as the duration from speech onset to speech offset using the above algorithm. We did not conduct any linguistic analysis of speech content. For the purpose of analysis, no distinction was made between the four conversation topics (i.e., data across topics was collapsed).

Two analyses are presented: (1) behaviour of triads in babble veersus speech-shaped noise to address the effects of acoustic environment complexity, (2) behaviour of dyads versus triads in speech-shaped noise to address the effects of interaction group complexity. For analysis, behaviours were separated into individual measures and group measures. Speech level, utterance duration, and orientation to the talker were individual measures, and were therefore averaged across individuals. Distance between individuals and gaps between turns were group measures, and hence were averaged across groups. To derive group measures in triads, measures were calculated between each pairing of individuals (i.e., person A and B, B and C, and C and A), then averaged to get a single score across each group for each condition.

The effect of auditory environment was analysed using a five (noise level) by two (noise type) repeated-measures ANOVA. The effect of conversation group size was analysed holding noise type constant. Hence, only behaviours recorded in speech-shaped noise conditions were analysed for triads, using a five (noise level) by two (group size) mixed ANOVA. In all cases where sphericity was violated, a Greenhouse-Geisser correction was used, and pairwise comparisons used Bonferroni corrections. Outliers (in which a participant’s mean in any condition was more than 3 standard deviations (SDs) from the mean of all participants) were removed. In the analysis of acoustic environment, this included two participants’ speech durations, and one participant’s head orientation, as they were greater than 3 SDs from the group mean (i.e., across all triads). In analysis of interaction group, this included six participants’ speech durations, as they were greater than 3 SDs from the group mean (i.e., across both dyads and triads).

Given the common focus on over 65-year-olds when addressing the importance of social interaction for older adults (Bowling et al., 2003), we ran additional exploratory analyses in which we split participants into groups of younger ( < 65 years; 14 in dyads and 20 in triads) and older (≥ 65 years; 16 in dyads and 13 in triads) participants. In these exploratory analyses we included age group as a between-subjects factor. Note that we only analyse effects of age group for the behaviours attributed to individuals (i.e., speech level, speech duration and head orientation), rather than behaviours of the group (i.e., inter-head distance).

## Results

First, we present results showing how conversation behaviour differs depending on the acoustic environment, by analysing the effect of noise level in triads for speech-shaped noise versus eight-talker babble. Then, we focus on how conversation behaviour differs depending on group size, by comparing behaviour of dyads and triads in speech-shaped noise.

### Effects of acoustic environment complexity

**Fig. 1:**
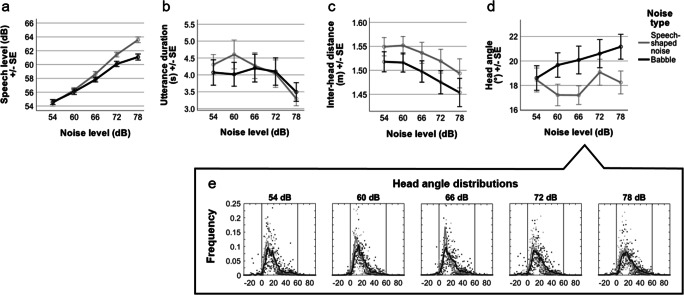
Plots **a–d** show mean triad behaviour by noise level and type, and plot **e** shows further detail of head angle (heavy lines for average histogram, dots for individual listeners). In **1a**, speech level showed an interaction between noise level and noise type, increasing more in speech-shaped than babble noise. In **1b**, utterance duration decreased with noise level similarly regardless of noise type. In **1c**, inter-head distance was generally smaller for babble than speech-shaped noise, and decreased with noise level. Finally, in **1d**, head angle was less directly oriented to the speaker in babble than speech-shaped noise, and was not affected by noise level

In terms of speech strategies, analysis of speech level (Fig. 1a) showed main effects of both noise level (F(2.60,83.11) = 285.84, *p* < .001) and noise type (F(1,32) = 18.07, *p* < .001). Critically, a significant interaction showed that as noise level increased, talkers increased their speech level more in speech-shaped noise than in babble (F(2.90,92.84) = 8.62, *p* < .001). Corrected post hoc tests showed this to be due to participants talking more loudly in speech-shaped noise than babble at higher noise levels: 66 dB (SSN = 58.7 dB; babble = 57.8 dB; *p* = .013), 72 dB (SSN = 61.5 dB, babble 60.1 dB; *p* < .001), and 78 dB (SSN = 63.6 dB, babble = 61.1 dB; *p* < .001). However, when analysing speech duration (Fig. 1b) there was only an effect of noise level (F(4,120) = 3.06, *p* = .019), showing that utterances were on average 792 ms longer in the quietest than loudest noise condition (54 dB = 4,187 ms; 78 dB = 3,395 ms; *p* = .042). For speech duration there was no impact of noise type (F(1,30) = 0.20, *p*>.65), and no interaction between noise level and noise type (F(4,120) = 0.61, *p* = .65).

In terms of movement strategies, analysis of distance between interlocutors (Fig. 1c) showed an effect of noise level, with interlocutors moving closer together as background noise increased (F(1,32) = 7.04, *p* = .012), and an effect of noise type, with interlocutors being on average 3.8 cm closer in the babble noise than in the speech-shaped noise (SSN = 153 cm; babble = 149.2 cm; F(1.45) = 25.72, *p* < .001). There was no interaction between noise level and noise type (F(2.32,74.16) = 0.550, *p* = .60). When analysing listeners’ head orientations (Figs. 1d and 1e) there was no effect of noise level (F(2.91, 90.15) = 2.24, *p* = .090). There was, however, an effect of noise type (F(1,31) = 6.79, *p* = .014), with the listener’s head being 2.0° less directly oriented towards the talker in babble than speech-shaped noise (SSN = 18.0°, babble = 20.0°). There was no interaction between noise level and noise type (F(2.79,86.46) = 1.96, *p* = .17).

In the exploratory analyses in which we included age group, all previously reported results for acoustic environment complexity remained significant. Only two additional effects were significant in these analyses. In the speech-duration analysis there was an additional main effect of age group (F(1,29) = 6.24, *p* = .018), whereby older participants produced utterances on average 1 s longer than younger participants (older = 4.65 s, younger = 3.65 s). In the analysis of head orientation there was also an additional main effect of age group (F(1,30) = 4.97, *p* = .034), whereby older participants oriented 3.1° less directly toward the speaker than younger participants (older = 20.9°, younger = 17.8°).

### Effects of interaction group complexity

**Fig. 2:**
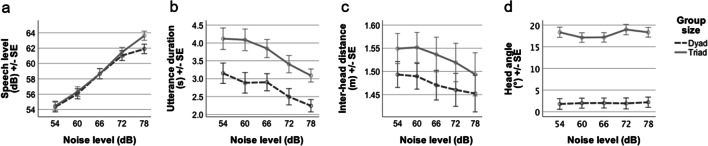
Behaviour of dyads (dotted) and triads (full) in speech-shaped noise at five different noise levels. In **2a**, speech level showed an interaction between noise level and group size, increasing more in triads than dyads. In **2b**, utterance duration was generally shorter for dyads than triads, and decreased with noise level. In **2c**, inter-head distance decreased with noise level similarly regardless of group size. Finally, in **2d**, head angle was less directly oriented to the speaker in triads than dyads, and was not affected by noise level

This analysis compared dyads and triads across the five noise levels in speech-shaped noise only. In terms of speech level (Fig. 2a), as expected there was an effect of noise level across both dyads and triads (F(2.93,178.40) = 624.22, *p* < .001), but there was no main effect of group size (F(1,61) = .36, *p* = .55). However, an interaction between noise level and group size showed that triads increased their vocal level in speech-shaped noise more than dyads (F(2.93,178.40) = 6.32, *p* < .001), with corrected post hoc tests showing that this was due to individuals talking 1.7 dB louder in triads than in dyads at 78 dB noise (SSN = 61.9 dB, babble = 63.6 dB; *p* = .05). At all other noise levels, speech levels in dyads and triads were indistinguishable. In terms of speech duration (Fig. 2b), there was an effect of noise level due to utterances being longer at low than at high noise levels (F(3.40,187.30) = 8.21, *p* < .001). An additional effect of group size showed that utterances were on average 974 ms longer in triads than in dyads (dyads = 2,735 ms, triads = 3,709 ms; F(1,55) = 14.59, *p* < .001). There was no interaction between noise level and group size (F(3.41,187.30) = 0.23, *p* = .90).

Regarding movement strategies, analysis of the distance between interlocutors (Fig. 2c) showed only an expected main effect of noise level (F(1.38,33.21) = 13.43, *p* < .001), with no main effect of or interaction with group size (main effect F(1,24) = 1.32, *p* = .26; interaction F(1.38,33.21) = 0.71, *p* = .45). Finally, in terms of head orientation (Fig. 2d), there was no effect of noise level (F(3.10,189.20) = 1.15, *p* = .33), but there was a main effect of group size (F(1,61) = 175.31, *p* < .001). Listeners in triads directed their heads on average 16.0° further from the talker than listeners in dyads (dyads = 2.0°, triads = 18.0°; see also Fig. 1e; F(1,61) = 175.31, *p* < .001). There was no interaction between noise level and group size (F(3.10,189.20) = 1.30, *p* = .28).

In the exploratory analysis in which we included age group, all previously reported results for interaction group complexity remained significant, and no additional effects were evident.

## Discussion

Many conversations in everyday life are in noisy environments. We often interact remarkably successfully in these situations, using a variety of different strategies to facilitate communication. We investigated how extra-linguistic behaviours change depending on the complexity of the acoustic environment and conversing group, and found an array of behaviours playing out differently depending on these factors. While we saw substantial support for our hypothesis that intelligibility-optimising behaviours would be enhanced in the more complex conversation contexts, we found that the specific behaviours chosen to facilitate communication depended on the manipulation. In detail, we showed increased vocal level, decreased interpersonal distance, and decreased utterance duration with noise level, but also key behavioural differences according to noise type. Specifically, we saw more optimal head orientation and distance between interlocutors in babble than speech-shaped noise, but a greater increase of vocal level in speech-shaped than babble noise. We also showed that when there are more than two interlocutors, listeners used more optimal head orientations and took greater advantage of gaining a speech turn by talking for longer. We note, however, that the behavioural adjustments we report are small, and interlocutors do not appear to spontaneously take full advantage of intelligibility-optimising strategies. These findings are critical to understand everyday communication and for effective technological development.

This is one of relatively few studies directly comparing interaction behaviour in different types of noise. We found different orientations of the listener to the talker in the two noise types; listeners in the more complex babble condition adopted a more optimal orientation than those in the less complex speech-shaped noise condition (optimal orientation modelled to be approximately 30°; Grange et al., 2018). We also saw interlocutors moving closer together as level increased in both noise types, but present the novel finding that interlocutors situated themselves several centimetres closer to their interaction partners overall in the punctate eight-talker babble compared to the diffuse speech-shaped noise, in spite of the same setup and condition order being counterbalanced. A possible explanation for the difference in proximity is that this movement towards the other interlocutors did not relate to the intelligibility of a partner’s speech directly, but rather was an indirect result of moving away from the competing sources of speech (i.e., the eight loudspeakers surrounding them). In fact, the difference between conditions only amounted to a 0.11 dB change in SNR (averaged across all noise levels). Given that the speech-shaped noise and babble differed both in being non-informational versus informational and diffuse versus punctate, future work could address the impact of speech versus spatial differences on behaviour.

However, while the above findings support the hypothesis that facilitatory extra-linguistic behaviours would be amplified in complex acoustic environments, we found an interaction between noise level and noise type, with talkers increasing their speech level more in louder levels of speech-shaped noise than eight-talker babble. While contrary to our hypotheses, this finding is similar to those of Garnier and colleagues comparing diffuse white noise and cocktail party noise (Garnier, Bailly, Dohen, & Welby, 2006). We suggest that this increased vocal level in the speech-shaped noise condition could relate to the inability to self-monitor in the higher levels of speech-shaped noise due to lack of temporal modulation (which would allow talkers to hear their vocal level in the gaps), as opposed to a behaviour adopted to enhance intelligibility. However, further study of vocal behaviour in modulated speech-shaped noise would be necessary to test this proposal.

In terms of group size, we showed that the presence of an additional listener led individuals to talk more loudly in the loudest noise levels, as hypothesised. We also found listeners to orient more optimally to the talker, though we note that it is not possible to confirm that this optimisation of head orientation definitely reflected an intelligibility-based adjustment as opposed to a social effect (of not wanting to exclude the other listener even when orienting to the talker). These differences in vocal level and orientation behaviours between dyads and triads support our hypothesis that intelligibility-optimising extra-linguistic behaviours would increase in more complex conversation situations. However, our findings relating to utterance duration are less clear. While an increased noise level caused utterance duration to decrease, likely reflecting talkers choosing to share less information when communication was challenging, an additional listener led utterance duration to increase. The latter effect, in which talkers produced longer utterances in triads than dyads, could either have reflected talkers speaking more slowly or producing more words. However, without analysis of the linguistic content to identify the number of words and thus duration of words we are not able to determine between these possibilities. Further work, combining automated extra-linguistic measurements with detailed linguistic analyses and qualitative investigation of speakers’ motivations, would be necessary to distinguish between such strategies.

The behavioural differences between dyads and triads highlight the difficulty of considering the dyad as a prototypical group. While substantial orientation and utterance length differences occurred with the addition of a third interlocutor into the group, we suggest that the effect of adding any further interlocutors would be markedly smaller. This is because most features of groups already occur in triads, but not all are possible in dyads. For example, the triad is the smallest unit that requires complex auditory attention switching between talkers, negotiation with other listeners when a talker surrenders the floor, and the potential for diverse listening roles (such as being the person who is being addressed, the person not being addressed, or being part of an addressed group). We therefore suggest that the triad may be a more appropriate minimal group construct when the goal is to extrapolate to larger group behaviour.

We also ran exploratory analyses in which we included age group to address whether the behaviours we report are specific to those typically considered ‘older adults’ (i.e., 65 years and over). While our participants were particularly highly functioning (self-selecting to regularly take part in research studies), and hence may not have demonstrated typical age-related cognitive decline, we nonetheless saw some differences in behaviour between the older and younger groups. Strikingly, older participants in the triadic conversations spoke for 1 s longer per utterance than the younger participants. We also found older participants in the triadic conversations to orient less directly to the talker, engaging a more beneficial orientation to the target speech. These effects indicate that some strategic behaviours may be engaged more strongly in older adults in complex interaction groups, but does not provide any evidence that the use of these strategies is specific to the noise environment (due to no interactions with noise level or noise type). Future work comparing the behaviour of younger and older interlocutors across a larger age range, as well as measuring potentially mediating cognitive skills, could shed further light on the impact of age on the use of these extra-linguistic strategies.

The studies we report addressed an array of conversation strategies across a variety of different modalities. We included contextual variables often not seen together, including features of the acoustic environment and features of the interaction, and used a relatively free task to address spontaneous conversation. Nonetheless, there are several limitations to this work. The conversation situation was not representative of everyday experience and the frequent noise-level changes may have affected strategy adoption (e.g., participants only minimally moving back from their partner in quiet due to how quickly the noise level could rise). Furthermore, recording behaviour from an intermediate condition of temporally modulated speech-shaped noise would have allowed us to better tie specific strategies to particular aspects of the noise (e.g., temporal modulation vs. information content). Finally, we investigated a group of older adults with broadly typical levels of age-related hearing loss. It is possible that some of our findings were driven by participants perceiving their conversation partner’s hearing difficulty, an issue that could be further investigated by manipulating hearing impairment within conversing groups.

In summary, here we have shown that people increase their adoption of several conversation strategies with the complexity of their auditory environment and interaction group. However, people do not exploit these strategies to their full potential, with the differences in use of intelligibility-optimising behaviours reported here providing only minor acoustic benefits. This raises the critical issue of how different strategies interrelate. We measured speech level, utterance duration, head orientation, and interpersonal distance, but a wide array of alternative strategies for improving intelligibility could have been engaged concurrently, such as gesture, lip-reading, or phonetic adjustments. To understand language use in real-life contexts, it is critical to begin to include measurement of such multimodal extra-linguistic behaviours, which, like language itself, appear to be context dependent.

The findings we report are important for both theoretical and practical reasons. On a theoretical level, the importance of extra-linguistic behaviours in conversation is beginning to be recognised, such as through the first theoretical model of multimodal binding in linguistic communication (Holler & Levinson, 2019). However, that model focuses on integration of modalities as opposed to the way multimodal extra-linguistic behaviours are adopted to communicate successfully. Our paper provides some of the necessary preparatory work to map this complex cognitive process. Furthermore, on a practical level, determining whether intelligibility-optimising behaviours are employed effectively in different contexts could inform tailored interventions for people that struggle in conversation, such as older adults or those with hearing loss. For example, by identifying behaviours that are not used to their full advantage (such as moving closer to a partner), it may be possible to develop training interventions to teach older individuals beneficial listening strategies. Alternatively, a better understanding of these extra-linguistic behaviours could allow hearing devices to be developed that work with the hearing-impaired user, by taking advantage of people’s natural intelligibility-optimising behaviours. Finally, such work could be used to inform interaction technologies, from contributing to the naturalness of avatars in virtual reality, to developing videoconferencing modes tailored to different acoustic environments or numbers of users.

### Open Practices Statement

Triad data have been deposited in the Open Science Framework repository, and can be accessed at: osf.io/w6t3y. Experiments were not preregistered.
